# Statistical ensemble analysis for simulating extrinsic noise-driven response in NF-κB signaling networks

**DOI:** 10.1186/1752-0509-7-45

**Published:** 2013-06-07

**Authors:** Jaewook Joo, Steven J Plimpton, Jean-Loup Faulon

**Affiliations:** 1 Department of Physics and Astronomy, University of Tennessee, Knoxville 37996, USA; 2 Scalable Algorithms Department, Sandia National Laboratories, Albuquerque, NM 87185, USA; 3 Department of Biology, Evry University, Evry Cedex, France

**Keywords:** Statistical ensemble, Extrinsic noise, Cell to cell variability, NF-κB signal transduction network

## Abstract

**Background:**

Gene expression profiles and protein dynamics in single cells have a large cell-to-cell variability due to intracellular noise. Intracellular fluctuations originate from two sources: *intrinsic* noise due to the probabilistic nature of biochemical reactions and *extrinsic* noise due to randomized interactions of the cell with other cellular systems or its environment. Presently, there is no systematic parameterization and modeling scheme to simulate cellular response at the single cell level in the presence of extrinsic noise.

**Results:**

In this paper, we propose a novel statistical ensemble method to simulate the distribution of heterogeneous cellular responses in single cells. We capture the effects of extrinsic noise by randomizing values of the model parameters. In this context, a statistical ensemble is a large number of system replicates, each with randomly sampled model parameters from biologically feasible intervals. We apply this statistical ensemble approach to the well-studied NF-κB signaling system. We predict several characteristic dynamic features of NF-κB response distributions; one of them is the dosage-dependent distribution of the first translocation time of NF-κB.

**Conclusion:**

The distributions of heterogeneous cellular responses that our statistical ensemble formulation generates reveal the effect of different cellular conditions, e.g., effects due to wild type versus mutant cells or between different dosages of external stimulants. Distributions generated in the presence of extrinsic noise yield valuable insight into underlying regulatory mechanisms, which are sometimes otherwise hidden.

## Background

Single cell imaging generated a surge of interest in the intracellular dynamics of biochemical species, uncovering significant cell-to-cell variations in gene expression [1-8] and protein dynamics [9,10]. This variability originates from intrinsic [1-8] and extrinsic noise [3,6,10] and critically affects cellular decision-making processes [9-13]. Moreover, cellular response averaged over a population of cells is oftentimes noticeably different from the responses of single cells. The variability in the latter contains rich information regarding the regulatory mechanisms in operation. Here, we present a novel computational method to predict the distribution of extrinsic noise-driven heterogeneous cellular responses and to unravel discrepancies between single-cell versus population-averaged responses.

**Figure 1:**
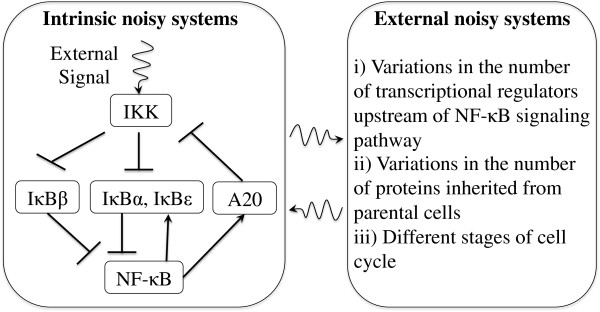
**Intrinsic and extrinsic noise as the source of the cell-to-cell variability in cellular responses in the NF-кB signaling networks.***Intrinsic* noise refers to the pure probabilistic nature of individual biochemical reactions in the signaling networks. *Extrinsic* noise refers to random interactions of the signaling networks with the external stochastic systems and originates from three sources: (i) fluctuating number of transcriptional regulators upstream of the signaling networks, (ii) fluctuating number of proteins inherited from parent cells, and (iii) different stages of their cell cycle.

Both intrinsic and extrinsic noise are the source of the large cell-to-cell variability in cellular responses [14]. *Intrinsic* noise refers to the pure probabilistic nature of individual biochemical reactions occurring within a cell. When the number of intracellular constituents is large, the cell’s behavior is well approximated by its expectation value according to the law of large numbers. But at the single-cell level, the number of molecules of certain species critical to a particular biochemical pathway can be small, and the range of statistical variation in the system needs to be considered [1-8]. *Extrinsic* noise refers to random interactions of the cell with other cells or its environment. Extrinsic fluctuations can originate from cells undergoing different stages of their cell cycle [15], fluctuations in the number of transcriptional regulators upstream of the signaling pathway of interest [3,6,9,10], and cell-to-cell variability in the copy number of proteins inherited from parent cells during cell division [10]. Extrinsic noise can affect the dynamics of cellular constituents locally in a specific signaling pathway or globally over the entire cell. In Figure 1, we summarize the effects of intrinsic and extrinsic fluctuations in the NF-κB signaling networks. The full effect of extrinsic noise should include “all” external stochastic effects that influence the cell, particularly the temporal fluctuations in the cellular kinetic conditions. However, in Ref. [10], Spencer et al. identified the most important source of extrinsic noise as the protein copy number inherited from the parent cell during cell division. Large cell-to-cell variations in the copy number of enzyme and regulatory protein could randomize the likelihood and the speed of any intracellular biochemical reaction. This means we can effectively “lump” all the effects of protein copy number variations into variations in kinetic rate constants. This is an attractive approach, because rate constants are an input into a variety of biochemical pathway modeling techniques.

A pathway modeling framework that uses deterministic or stochastic differential equation models requires *a priori* knowledge of the structure of the biochemical reaction network, mathematical functional forms for the biochemical reactions, and associated reaction rate constants. Since limited or incomplete information is often all that is available to modelers, a computational model is often parameterized by using a nonlinear fitting algorithm. A conventional parameterization scheme identifies a single set of kinetic parameter values by minimizing the χ^2^ distance between experimental data and a prediction made by the model. Sloppy Cell and other similar parameterization algorithms include experimental errors in the parameterization by fitting to a rather large experimental error bar [16]. But both conventional and Sloppy Cell parameterization schemes assume a deterministic and homogeneous biological response to a stimulus and aren’t designed to handle the heterogeneous, stochastic behavior of single cells and its dependence on extrinsic noise.

In order to capture extrinsic noise and its effect on intracellular response, we propose a novel parameterization method, the “statistical ensemble” (SE) scheme, named after a key concept in statistical physics [17]. A cell is regarded as a complex system comprising a large number of components and elementary interactions among them. A population of cells consists of a large number of replicates, each with different microscopic intracellular states. The statistical ensemble average, or macroscopic observable, is equated with the cellular response averaged over the population of cells. The ensemble is generated by assigning randomly sampled values of kinetic rate constants and copy numbers of regulatory proteins to each cell in the ensemble. All other external noisy systems that interact with the cell, but which are not modeled explicitly, are treated as extrinsic noise. The effect of the noise is included in the sampling that produces the randomized microscopic state of each cell in the ensemble.

A key point is that the resulting dynamic response of the ensemble of cells is no longer a single output but is a distribution of heterogeneous responses. Each response can be computed independently, which allows for parallelism in the computation. An equal weight is assigned to the response from each replicate, to calculate the ensemble averaged cellular response. The SE scheme thus enables modeling of the irregular, dissimilar, and diverse individual cellular behaviors while reproducing the macroscopically observable population-level response.

In the most general sense, the success of the SE scheme depends on identifying and characterizing the biologically correct distribution of extrinsic noise for the system of interest, so that its effects can be encoded in the random sampling of cellular microstates. In this work, we use experimental population-level data to parameterize the range of feasible kinetic rate constants and copy numbers of specific molecules, and then sample uniformly around the mid-point of the range to generate cellular microstates. We illustrate the power of even this simplified SE approach for modeling the NF-кB signaling system.

NF-кB is a pleiotropic regulator of gene control and plays significant roles in various cellular functions such as differentiation of immune cells, development of lymphoid organs, and immune activation [18-20]. NF-кB shuttling between the nucleus and cytoplasm is auto-regulated by the NF-кB signaling module, which consists of IкB (inhibitor кB), IKK (IкB kinase), and NF-кB. In the absence of stimulus, IкB forms a hetero-dimeric complex with NF-кB, preventing NF-кB from entering into the nucleus. Upon stimulation, phosphorylated IKK catalyses the degradation of IкB from the IкB-NF-кB complex and frees up NF-кB whose nuclear localization initiates transcription of NF-кB target genes such as inflammatory cytokines (TNFα, IL-1, IL-6), chemotactic cytokines (MIP-1a), Th1 and Th2 response activation (IFN and IL-10), and lastly, but most importantly, negative regulators (IкBα, IкBβ, IкBϵ, and A20) which terminate the NF-кB signaling. Based on current knowledge of NF-кB signaling, Hoffmann *et al.* constructed a complex biochemical reaction network for the NF-кB signaling pathway consisting of IKK, NF-кB, and three IкB isoforms and transformed it into a set of ordinary differential equations with dozens of unknown kinetic parameter values [21]. After identifying a single set of parameter values yielding the best fit of the model to population level experimental data, they used their model to elucidate the role of each of three IкB isoforms: IкBα induces oscillatory shuttling of NF-кB while IкBβ and IкBϵ damp the oscillations [21]. Lipniacki *et al.* extended the model, showing that an additional negative regulator A20 has a definitive role as a NF-кB signal terminator, by deactivating IKK phosphorylation [22-24]. Using fluorescence microscopy, Nelson *et al.* and several other groups showed a remarkably heterogeneous intracellular response for this signaling network at the single-cell level; some cells exhibited sustained oscillatory shuttling of NF-кB while others exhibited non-oscillatory behavior [25-33].

**Figure 2:**
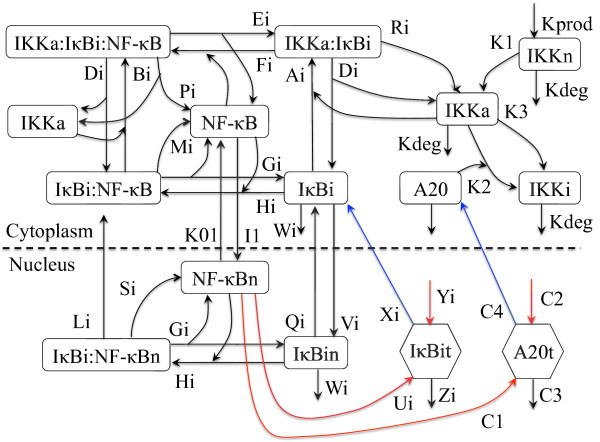
**Biochemical network model for the IKK-IкB-NF-кB-A20 signaling module.** Top panel: A schematic description of our comprehensive model for NF-кB signaling. The arrows indicate activation and the perpendicular lines denote inhibition. Bottom panel: the model consists of IKK (IкB kinase), IкB isoforms (IкB*i, i* = α, β, ϵ), and A20. NF-кBn and IкB*i*n denote their nuclear components. Squares are for proteins; hexagons are for mRNA. Black arrows indicate either association or dissociation or degradation of proteins; red (blue) arrows denote mRNA (protein) synthesis.

In this paper, we model extrinsic noise via randomization of the kinetic parameters of the IKK-NF-кB-IкB-A20 signaling system and predict several distributions of dynamical NF-кB responses. The signaling network we model is shown in Figure 2 and consists of IKK, cytoplasmic and nuclear NF-кB, and two groups of negative regulators (three isoforms of IкB and A20). Using the statistical ensemble (SE) scheme, we demonstrate that extrinsic noise, modeled as fluctuations in kinetic parameter values, can generate the observed experimental population-level response as the SE average, as well as a heterogeneous distribution of individual cellular responses. In section Results.A we show that the SE average of key biochemical species concentrations in the NF-кB signaling network can be accurately fit to experimental population-level data for wild type and various mutant cases. In section Results.B, we predict the distributions of various dynamic characteristics of NF-кB cellular responses. In section Results.C, we make a prediction about dosage-dependent NF-кB responses in single cells, i.e., the dosage-dependent distribution of various NF-кB dynamic characteristics in individual cells. Lastly, in section Results.D, we predict that both dose-response curves from individual cells and their SE average are sigmoidally shaped.

## Results

### Statistical ensemble average of key biochemical species concentrations in the NF-кB signaling network is fit to experimental population-level data

#### The wild type case

**Figure 3:**
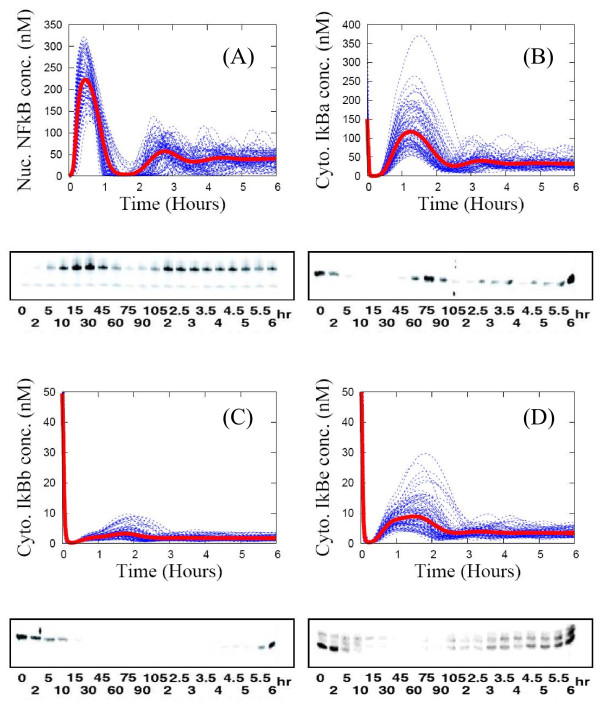
**Individual time-series curves (blue lines) and the ensemble average (red line) of key protein concentration for an ensemble of 1000 replicates of the wild type NF-кB signaling system.** Computational results are compared side-by-side with population-level experimental data from Ref. [20]. Panel (**A**): nuclear concentration of NF-кB. Panels (**B**), (**C**), (**D**) are respectively cytoplasmic concentrations of IкBα, IкBβ, and IкBϵ proteins.

**Figure 4:**
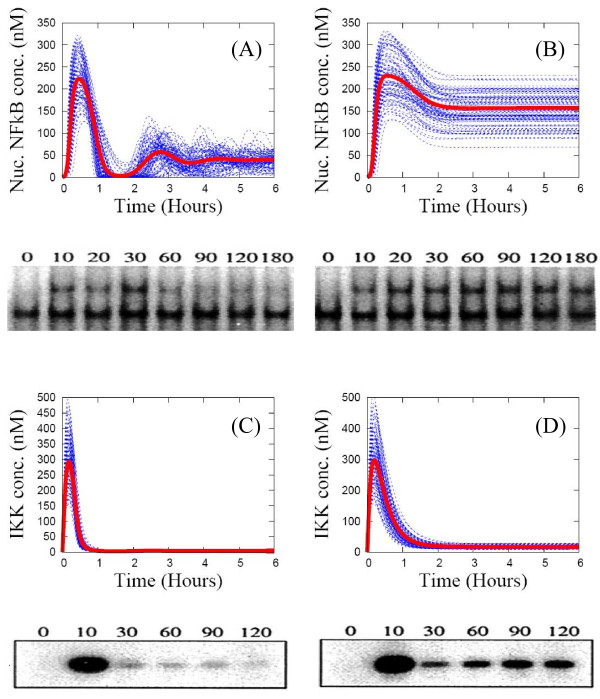
**Individual time-series curves (blue lines) and the ensemble average (red line) of key protein concentrations are obtained for an ensemble of 1000 replicates of a wild type (A, C) and an A20 knocked-out mutant (B, D).** Computational results are compared with population-level experimental data from Ref. [36]. Top panels: nuclear concentration of NF-кB. Bottom panel: IKK concentration.

For this reaction pathway, the statistical ensemble (SE) scheme generates significant cell-to-cell variability in protein dynamics. Yet the SE averages agree well with population-level experimental data (Electro Mobility Shift Assay (EMSA) or western blot) for key biochemical species concentrations as shown in Figure 3. For the nuclear NF-кB profiles in Figure 3(A), the first translocation times (timing of the first peak) of the individual NF-кB profiles (in blue) are almost identical, while the first maxima (amplitude of the first peak) vary significantly with a variance up to 100% of the SE average (in red). However, both the timings and amplitudes of subsequent peaks exhibit significant cell-to-cell variability. Consequently the SE average is a strongly damped oscillatory pattern with rapid decay of subsequent peak amplitudes. Thus, the effect of extrinsic noise on this observable is a “masking effect of averaging over a population of asynchronous curves”, just as for intrinsic noise [34]. The large variation in the first-peak amplitude of nuclear NF-кB concentration in Figure 3(A) originates from the IKK profile in Figure 4(C), where the IKK concentration time courses from individual cells also exhibit significant differences in their first maximum. This induces large variation in the first minimum of IкB isoforms as shown in Figures 3(B)-(D). Thus, the cell-to-cell variation in kinetic rate constants regulating the levels of both pre-activated IKK (IKKn) and activated IKK (IKKa) is the source of similar variation in the first maximum of nuclear NF-кB concentration [35]. Likewise, the asynchronous behavior of the individual nuclear NF-кB profiles after two hours, as shown in Figure 3(A), originates from the cell-to-cell variability in the second-peak amplitude of the IкB isoforms in Figures 3(B)-(D).

#### The mutant case - double knocked-out IкB isoforms and knocked-out A20

**Figure 5:**
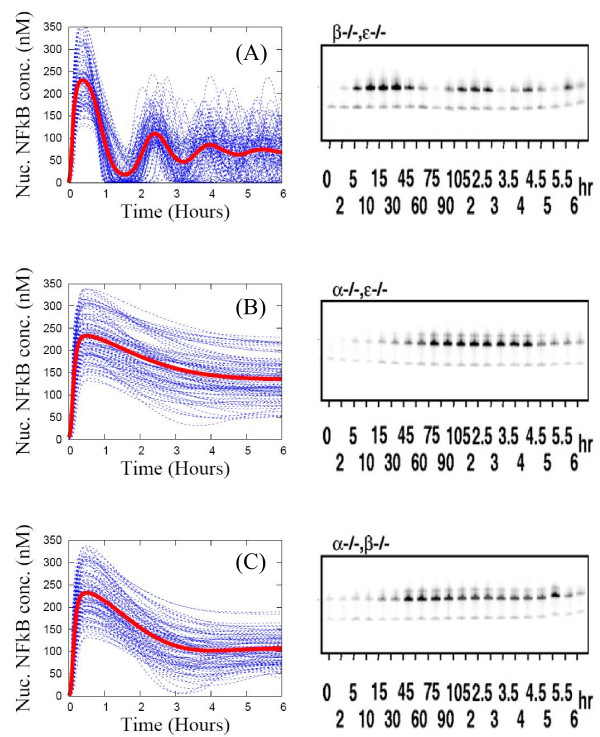
**Individual time-series curves (blue lines) and the ensemble average (red line) of key protein concentrations for an ensemble of 1000 replicates of a IкB double gene knocked-out mutant.** Computational results (left column) are compared with population-level experimental data (right column) from Ref. [20]. Panel (**A**): IкBβ and IкBϵ knocked-out mutant. Panel (**B**): IкBα and IкBβ knocked-out mutant. Panel (**C**): IкBα and IкBϵ knocked-out mutant.

**Figure 6:**
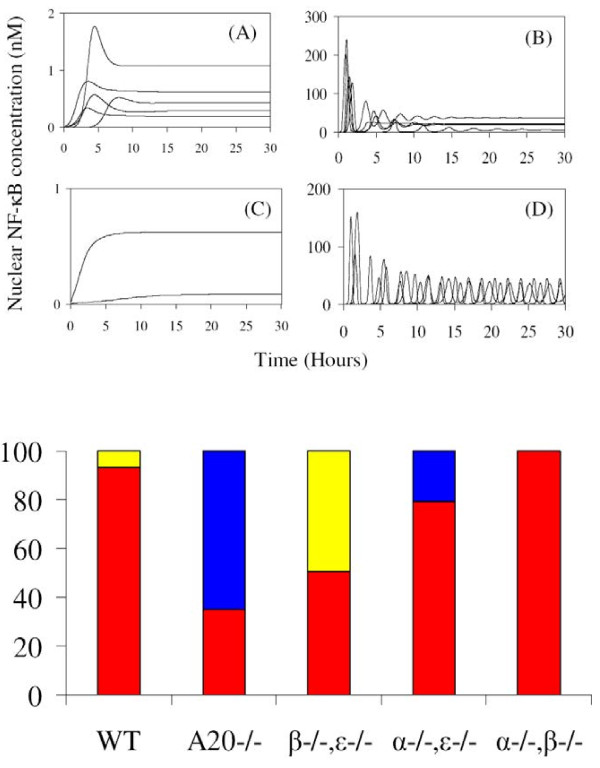
**Distributions of four dynamic patterns of the individual time-series curves of nuclear NF-кB profiles for an ensemble of 1,000 replicates of the wild type, A20 knocked-out mutant, and three IкB genes double knocked-out mutants.** A few examples of four dynamic patterns are plotted in the top panel: (**A**) single-peaked pattern (blue), (**B**) under-damped oscillation (red), (**C**) hyperbolic pattern (black), and (**D**) sustained oscillation (yellow) where color within a parenthesis denotes color in the bottom panel. Individual time-series curves are classified as one of the four dynamic patterns.

To simulate the dynamics of mutants, we set the mRNA synthesis rates for two of the three IкB isoforms and A20 to zero. For the IкBβ and IкBϵ knocked-out mutant shown in Figure 5(A), the peaks of the SE average correspond closely to the peaks of population-level experimental data (EMSA) at 15 min, 2.5 hours, 4 hours, and 5.5 hours. The individual profiles of nuclear NF-кB concentration are much more oscillatory (about half the curves exhibit sustained oscillations as shown in Figure 6) than for the wild type data (only 10% are sustained oscillations in Figure 6). But, the SE average of this mutant is a damped oscillatory pattern, with a bit more dynamic variation than that of the wild type. This is again mainly due to “the masking effect of averaging over a population of asynchronous curves”. For the IкBα and IкBϵ knocked-out and the IкBα and IкBβ knocked-out mutants shown in Figure 5(B) and 5(C), the SE averages of nuclear NF-кB show a “single-peaked” pattern similar to the population-level EMSA data, though the timings of the peaks differ by 1 hour. The single-peak amplitudes vary significantly with a variance as large as 100% of the SE average. For the A20 knocked-out mutant in Figure 4(B) and 4(D), both the SE averages of nuclear NF-кB and IKK profiles exhibit single-peaked patterns in good agreement with the population-level experimental data. Again, the individual nuclear NF-кB profiles differ significantly. For all the mutants, though their SE averages for nuclear NF-кB profiles exhibit simple dynamic patterns, the cell-to-cell variability is large due when extrinsic noise is included in the model.

#### Dependence of SE average on heterogeneity

**Figure 7:**
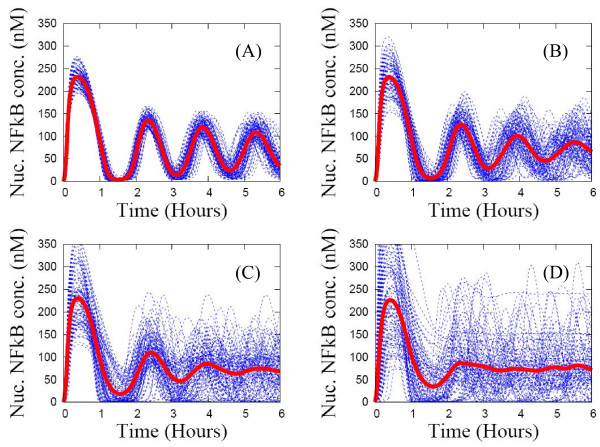
**Dependence of the individual time-series curves (blue lines) and the statistical ensemble average (red line) of nuclear NF-кB profiles for a mutant with IкBβ and IкBϵ genes double knocked-out, on the heterogeneity factor χ (the interval size of the uniform distribution or kinetic rate parameters).** (**A**) χ = 10% ; (**B**) χ = 30% ; (**C**) χ = 50% ; (**D**) χ = 70%.

In Figure 7 we show how to use population-level experimental data as a constraint when choosing a heterogeneity factor χ, defined as the interval size of the uniform distribution from which kinetic rate constants are sampled, as inputs to the pathway model. Centering the kinetic rate constants at their reference values, we vary χ and observe how heterogeneous the individual cell profiles of nuclear NF-кB become. Note that the SE average of nuclear NF-кB becomes less oscillatory for higher values of χ in Figure 7. For a small χ = 10% in Figure 7(A), all individual curves remain in phase with each other, making the SE average also highly oscillatory. For higher values of χ = 50% and χ = 70% in Figures 7(C) (χ = 50%) and 7(d) (χ = 70%), a large fraction of individual curves are sustained oscillations, but quickly become out of phase, resulting in an SE average that is strongly under-damped. Because higher χ values cover a larger sampling space, individual nuclear NF-кB curves bifurcate into different classes of patterns: some are sustained oscillatory while others are single-peaked. Thus, if the population-level experimental data exhibit sustained oscillations versus damped oscillations versus single-peak profiles, the variation in single-cell profiles induced by χ can be used to guide sampling from an appropriate range of heterogeneity when generating input rate constants.

In this subsection, we showed how the SE method with its many replicates is a model for a population of cells in a heterogeneous set of intracellular states. By varying the heterogeneity factor χ for sampling kinetic parameters used as inputs to the pathway model, fits to experimental data can be produced even when population-averaged data and single-cell data exhibit different characteristics, as in the NF-кB signaling system. In the next subsection, we discuss the distribution of single-cell NF-кB responses in more detail.

### Prediction of distributions of individual cellular responses for the wild type and mutants

#### Distributions of dynamic features

**Figure 8:**
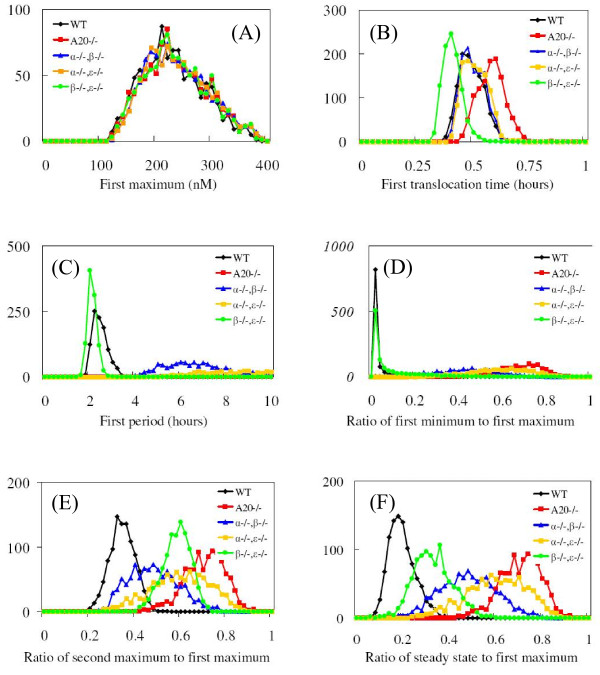
**Distributions of six dynamic features of nuclear NF-кB profiles, for an ensemble of 1,000 replicates of the wild type (black lines), A20 knocked-out mutant (red lines), IкBα and IкBβ genes double knocked-out mutant (blue lines), IкBα and IкBϵ genes double knocked-out mutant (yellow lines), and IкBβ and IкBϵ genes double knocked-out mutants (green lines).** The six dynamic features are First Maximum (the amplitude of the first peak) in panel (**A**), First Translocation Time (the timing of the first peak) in panel (**B**), First Period (the time between the first and the second peaks) in panel (**C**), Ratio of First Minimum to First Maximum (ratio of the first minimum value to the first maximum value) in panel (**D**), Ratio of Second Maximum to First Maximum (ratio of the second peak amplitude to the first maximum value) in panel (**E**), and Ratio of Steady State to First Maximum (ratio of the steady state level to the first maximum value) in panel (**F**).

In Figure 8, we summarize the output of our SE computational model for distributions of single-cell responses for the wild type and the mutants discussed in the previous sub-section. Six dynamic features are shown: the amplitude of the first peak (First Maximum), the timing of the first peak (First Translocation Time), the time between the first and the second peaks (First Period), the level of the first minimum (First Minimum), the amplitude of the second peak (Second Maximum), and the asymptotic Steady State value. Surprisingly, for each dynamic feature, there is a significant amount of overlap between the distributions for the wild type and those of the mutants. This implies that if we used a conventional modeling scheme which fits a single set of parameter values and outputs a single representative time-series of intracellular response, we could draw incorrect conclusions as to the effect of a knocked-out gene on cellular response. To avoid this, we compute the entire distribution of responses and look for significant changes when genes are knocked out. In Figure 8(A), the distributions of the First Maximum are the same for both the mutants and the wild type. This dynamic feature is thus not an indicator of the physiological defects caused by the knock-out genes. In Figure 8(B), the distribution of the First Translocation is shifted to the right for the A20 knocked-out mutant and to the left for IкBβ and IкBϵ double knocked-out mutant, whereas the wild type and two other mutants have similar distribution. In Figure 8(C), only the wild type and the IкBβ and IкBϵ double knocked-out mutant have well-defined periods of roughly 2 hours; the First period of other mutants is too broadly distributed to define an average. In Figure 8(D), the ratio of the First Minimum to the First Maximum indirectly measures the spikiness of the oscillations; the smaller the ratio, the spikier the temporal profile becomes. Only the wild type and the IкBβ and IкBϵ double knocked-out mutant exhibit a spiky response. In Figure 6(F), the ratio of the Steady State to the First Maximum provides useful information about the relative magnitude and strength of the negative regulators of IкB isoforms and A20. Since the distributions of the First Maximum are the same for the wild type and mutants, we conclude that the smaller steady-state level of nuclear NF-кB concentration infers stronger negative feedback. The mutants ordered by steady-state level are as follows: A20 knocked-out mutant < IкBα and IкBϵ knocked-out mutant < IкBα and IкBβ knocked-out mutant < IкBβ and IкBϵ knocked-out mutant < wild type. The relative strength of the negative regulators can then be inferred: A20 > IкBα > IкBϵ > IкBβ. Of course, this ordering is consistent with the choice of nominal values for the respective kinetic rate constants, as listed in Table 1.

**Table 1:** Biochemical reactions and their associated reaction rate constants in the computational model of the NF-κB signaling network

**Reactions**	**I**	**II**	**III**	**IV**	**V**
IKKa + IкBα → IKKa:IкBα	Aα	[a]	0.2	[1]	0.1813
IKKa + IкBβ → IKKa:IкBβ	Aβ	[a]	0.05	[3]	0.02997
IKKa + IкBϵ → IKKa:IкBϵ	Aϵ	[a]	0.05	[3]	0.04244
IKKa + IkBα:NF-кB → IKKa:IкBα:NF-кB	Bα	[a]	1	[1]	1.024
IKKa + IkBβ:NF-кB → IKKa:IкBβ:NF-кB	Bβ	[a]	0.25	[3]	0.3683
IKKa + IkBϵ:NF-кB → IKKa:IкBϵ:NF-кB	Bϵ	[a]	0.25	[3]	0.42
NF-кBn → NF-кBn + A20t	C1	[b]	0.0000005	[1]	0.000000506
0 → A20t	C2	[c]	0	[1]	0
A20t → 0	C3	[b]	0.0004	[1]	0.0002438
A20t → A20t + A20	C4	[b]	0.5	[1]	0.5807
A20 → 0	C5	[b]	0.0003	[1]	0.0003769
IKKa:IкBα → IKKa + IкBα	Dα	[b]	0.00125	[2]	0.002046
IKKa:IкBβ → IKKa + IкBβ	Dβ	[b]	0.00175	[2]	0.0005609
IKKa:IкBϵ → IKKa + IкBϵ	Dϵ	[b]	0.00175	[2]	0.002142
IKKa:IкBα:NF-кB → IKKa + IккBα:NF-кB	Dα	[b]	0.00125	[2]	0.002046
IKKa:IkBβ:NF-кB → IKKa + IкBβ:NF-кB	Dβ	[b]	0.00175	[2]	0.000561
IKKa:IкBϵ:NF-кB → IKKa + IкBϵ:NF-кB	Dϵ	[b]	0.00175	[2]	0.002142
IKKa:IкBα:NF-кB → IKKa:IкBα + NF-кB	Eα	[b]	0.000001	[2]	0.00000144
IKKa:IкBβ:NF-кB → IKKa:IкBβ + NF-кB	Eβ	[b]	0.000001	[2]	0.00000124
IKKa:IкBϵ:NF-кB → IKKa:IкBϵ + NF-кB	Eϵ	[b]	0.000001	[2]	0.00000064
IKKa:IкBα + NF-кB → IKKa:IкBα:NF-кB	Fα	[a]	0.5	[2]	0.3789
IKKa:IкBβ + NF-кB → IKKa:IкBβ:NF-кB	Fβ	[a]	0.5	[2]	0.2135
IKKa:IкBϵ + NF-кB → IKKa:IкBϵ:NF-кB	Fϵ	[a]	0.5	[2]	0.3528
IкBα:NF-кB → NF-кB + IкBα	Gα	[b]	0.000001	[2]	0.00000064
IкBβ:NF-кB → NF-кB + IкBβ	Gβ	[b]	0.000001	[2]	0.00000044
IкBϵ:NF-кB → NF-кB + IкBϵ	Gϵ	[b]	0.000001	[2]	0.00000069
IкBαn:NF-кBn → NF-кBn + IкBαn	Gα	[b]	0.000001	[2]	0.00000064
IкBβn:NF-кBn → NF-кBn + IкBβn	Gβ	[b]	0.000001	[2]	0.00000044
IкBϵn:NF-кBn → NF-кBn + IкBϵn	Gϵ	[b]	0.000001	[2]	0.00000069
IкBα + NF-кB → IкBα:NF-кB	Hα	[a]	0.5	[2]	0.4593
IкBβ + NF-кB → IкBβ:NF-кB	Hβ	[a]	0.5	[2]	0.7753
IкBϵ + NF-кB → IкBϵ:NF-кB	Hϵ	[a]	0.5	[2]	0.2895
IкBαn + NF-кBn → IкBαn:NF-кBn	Hα	[a]	0.5	[2]	0.4593
IкBβn + NF-кBn → IкBβn:NF-кBn	Hβ	[a]	0.5	[2]	0.7753
IкBϵn + NF-кBn → IкBϵn:NF-кBn	Hϵ	[a]	0.5	[2]	0.2895
NF-кB → NF-кBn	I1	[b]	0.0025	[1]	0.003037
NF-кBn → NF-кB	K01	[b]	0.00005	[3]	0.00005537
IKKn → IKKa	K1	[b]	0.0025	[1]	0.003273
A20 + IKKa → A20 + IKKi	K2	[a]	0.1	[1]	0.07075
IKKa → IKKi	K3	[b]	0.0015	[1]	0.00202
0 → IKKn	Kprod	[c]	0.000025	[1]	0.000009752
IKKn, IKKa, or IKKi → 0	Kdeg	[b]	0.000125	[1]	0.0001561
Volume ratio of cytoplasm to nucleus	Kv	1	5	[1]	5
IкBαn:NF-кBn → IкBα:NF-кB	Lα	[b]	0.01	[1]	0.013979
IкBβn:NF-кBn → IкBβ:NF-кB	Lβ	[b]	0.005	[3]	0.001567
IкBϵn:NF-кBn → IкBϵ:NF-кB	Lϵ	[b]	0.005	[3]	0.006583
IкBα:NF-кB → NF-кB	Mα	[b]	0.000025	[1]	0.00002837
IкBβ:NF-кB → NF-кB	Mβ	[b]	0.000025	[3]	0.00003609
IкBϵ:NF-кB → NF-кB	Mϵ	[b]	0.000025	[3]	0.00000866
Total NF-кB concentration	NF-кB	[d]	0.06	[1]	0.06
IKKa:IкBα:NF-кB → IKKa + NF-кB	Pα	[b]	0.1	[1]	0.12928
IKKa:IкBβ:NF-кB → IKKa + NF-кB	Pβ	[b]	0.05	[3]	0.06454
IKKa:IкBϵ:NF-кB → IKKa + NF-кB	Pϵ	[b]	0.05	[3]	0.08434
IкBαn → IкBα	Qα	[b]	0.0005	[1]	0.0005123
IкBβn → IкBβ	Qβ	[b]	0.0005	[3]	0.0007398
IkBϵn → IkBϵ	Qϵ	[b]	0.0005	[3]	0.0002184
IKKa:IкBα → IKKa	Rα	[b]	0.1	[1]	0.123
IKKa:IкBβ → IKKa	Rβ	[b]	0.1	[3]	0.03837
IKKa:IкBϵ → IKKa	Rϵ	[b]	0.1	[3]	0.1571
IкBαn:NF-кBn → NF-кBn	Sα	[b]	0.000001	[2]	0.00000037
IкBβn:NF-кBn → NF-кBn	Sβ	[b]	0.000001	[2]	0.000001131
IкBϵn:NF-кBn → NF-кBn	Sϵ	[b]	0.000001	[2]	0.000001037
NF-кBn → NF-кBn + IкBαt	Uα	[b]	0.0000005	[1]	0.000000279
NF-кBn → NF-кBn + IкBβt	Uβ	[b]	0	[2]	0
NF-кBn → NF-кBn + IкBϵt	Uϵ	[b]	0.00000005	[3]	0.000000059
IкBα → IкBαn	Vα	[b]	0.001	[1]	0.0009786
IкBβ → IкBβn	Vβ	[b]	0.001	[3]	0.0004871
IkBϵ → IkBϵn	Vϵ	[b]	0.001	[3]	0.00147
IкBα, IкBαn → 0	Wα	[b]	0.0001	[1]	0.000132
IкBβ, IкBβn → 0	Wβ	[b]	0.0001	[3]	0.000133
IкBϵ, IкBϵn → 0	Wϵ	[b]	0.0001	[3]	0.000042
IкBαt → IkBαt + IkBα	Xα	[b]	0.5	[1]	0.4552
IкBβt → IкBαt + IкBβ	Xβ	[b]	0.5	[3]	0.3828
IкBϵt → IкBαt + IкBϵ	Xϵ	[b]	0.5	[3]	0.3304
0 → IкBαt	Yα	[c]	0.00000005	[3]	0.000000084
0 → IкBβt	Yβ	[c]	0.000000005	[3]	0.00000000414
0 → IкBϵt	Yϵ	[c]	0.000000005	[3]	0.00000000508
IкBαt → 0	Zα	[b]	0.0004	[1]	0.0003375
IкBβt → 0	Zβ	[b]	0.0004	[3]	0.0002031
IкBϵt → 0	Zϵ	[b]	0.0004	[3]	0.0004742

The far left column denotes biochemical reactions. The symbols for reaction rate constants are in the column I. Their units and their published nominal values are in columns II and III respectively. Column IV denotes the reference: [1] for Ref. [21], [2] for Ref. [20], and [3] for the average of values from Refs. [20,21]. Column V lists values used in this paper. The units for [a] are μM^-1^ s^-1^, for [b] are s^-1^, for [c] are μM s^-1^, and for [d] are μM.

#### Distribution of dynamic patterns

The individual time-series of the nuclear NF-кB concentrations can be classified into one of four dynamic patterns (damped oscillation, sustained oscillation, single peaked, and monotonic-increasing patterns) as shown in Figure 6. The underlying mechanism for each dynamic pattern is rather simple. The monotonic-increasing (or over-damped) pattern originates from strong negative feedback loops, while the single-peaked pattern results from weak negative feedback loops. The oscillatory patterns arise from intermediate-strength negative feedback loops. But it remains an open question to correlate each dynamic pattern with a specific cellular physiology [37-39]. To elucidate this connection, we stimulate the ensemble of NF-кB signaling networks with the same signal strength (TR = 1), for both the wild type and mutants. We then classify a thousand individual temporal profiles into one of the four dynamic patterns. The distributions of the patterns are represented by bar graphs in Figure 6 which shows that both the wild type and mutants exhibit at least two different patterns of response under the same strength of stimulation. For the wild type, most of the nuclear NF-кB profiles have a damped-oscillatory pattern, with less than 10% of the profiles as sustained-oscillatory. This indicates a damped-oscillatory response is the most probable, and it is robust against perturbation of the network parameter values. For the mutant with a knocked-out A20 gene, both single-peaked and damped-oscillatory patterns are nearly equally probable. But the damped oscillatory profiles are very similar to a single-peaked pattern. Thus for this mutant, a damped-oscillatory response occurs in a region of the parameter space where the negative regulators are not strong enough to induce the oscillatory pattern. For the mutant with IкBβ and IкBϵ genes double knocked-out, sustained-oscillatory and damped-oscillatory patterns are equally probable responses. The damped-oscillatory responses in this mutant are very different from those in the mutant with a knocked-out A20 gene, and are more similar to a sustained oscillation. The fraction of sustained-oscillatory responses (about 50%) dramatically increases in comparison to the wild type case (less than 10%). For mutants with IкBα and IкBβ genes double knocked-out and with IкBα and IкBϵ genes double knocked-out, their respective distributions are similar to that of the mutant with the A20 gene knocked-out. As shown in Figures 5(B) and 5(C) and Figure 4(B), both the individual profiles and the statistical ensemble average of the nuclear NF-кB concentrations for all these mutants (A20 gene knocked-out, IкBα and IкBβ genes double knocked-out, IкBα and IкBϵ genes double knocked-out) exhibit similar single-peaked patterns. In summary, there are two distinctive groups exhibiting two respective patterns of nuclear NF-кB profile response: the first group, consisting of the wild type and the IкBβ and IкBϵ double knocked-out mutant, is dominated by highly oscillatory responses. The second group, consisting of the A20 knocked-out, the IкBα and IкBβ double knocked-out, and the IкBα and IкBϵ double knocked-out mutants, shows mostly single-peaked (non-oscillatory) responses.

In this subsection, we used the SE method to generate distributions of various dynamical responses from a large ensemble of single-cell simulations, and compared those distributions for the wild type and various mutants. We made two key findings. First, there is significant overlap between the distributions of the wild type and mutants. This indicates that two individual cells, even if they are genetically different, can respond to the same stimulus in a similar manner. A better way to characterize the differences induced by the differing genetic conditions is to model a large ensemble of cells and compare the full distributions of single-cell responses. Second, for this biochemical pathway, we observed that distributions of the first Maximum response were the same for any genetic conditions. Similarly, the distributions of the first translocation time responses were the same for the wild type and two of the genetic mutants. This means that some dynamic features are not good indicators of changes in the NF-кB signaling system for genetic comparative studies. The SE approach can be used to screen out bad indicators among the many possible candidates. In the next subsection, we investigate the distributions of dynamic responses for the NF-кB signaling system under two different dosage conditions.

### Statistical ensemble analysis of dosage-dependent NF-кB dynamical behavior

#### Dosage-dependent dynamical behaviors of individual and ensemble-averaged temporal profiles

**Figure 9:**
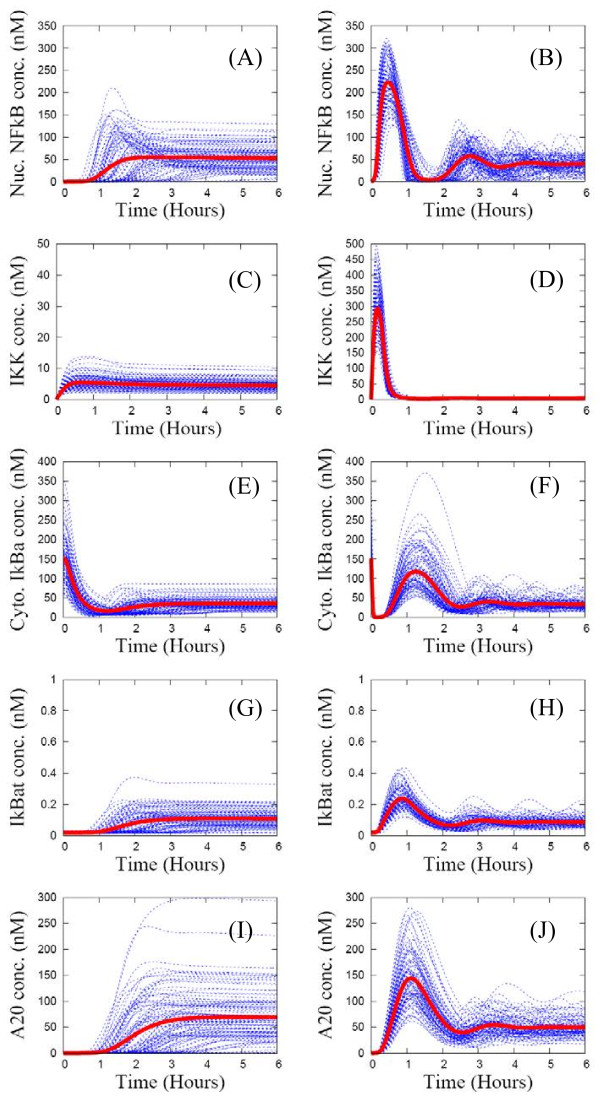
Individual time-series curves (blue lines) and the ensemble average (red) of the key protein concentrations for an ensemble of 1000 replicates of the wild type, stimulated by small dosage (A, C, E, G, and I) or large dosage (B, D, F, H, and J).

We numerically investigated the NF-кB signaling network in response to two different stimulation dosages. As shown in Figure 9, even though a small dosage (TR = 0.01) is 100 times smaller than a large dosage (TR = 1), the small dosage still triggers a substantial amount of NF-кB response from about half the replicates. The other half do not respond at all to the small dosage. In contrast, the large dosage induces strong NF-кB response from all the replicates homogeneously. For example, in Figure 9(A) where the ensemble receives a small dosage, half the temporal profiles of nuclear NF-кB have a single peak and the other half do not. For the half with a peak, the peaks occur after hours of time-delay and there is a large variation in the delays. The steady-state level of nuclear NF-кB concentration is broadly distributed between zero and 100 nM. In Figure 9(B), the large dosage induces a synchronized appearance of the first peak in all the temporal profiles after a time delay of half an hour. However, even with a large dosage, there is a large cell-to-cell variation both in the amplitude of the first peak and in the timing of successive peaks. In Figures 9(C) and (D), IKK responses to the small dosage stimulation are sharply different from those with large dosage stimulation, i.e., very low levels of IKK versus single-peaked responses with a large amplitude. These IKK profiles are inversely correlated with the profiles of cytoplasmic IкBα. The steady state levels of A20 are 2-3 times higher.

#### Dosage-dependent distribution of the dynamic features

**Figure 10:**
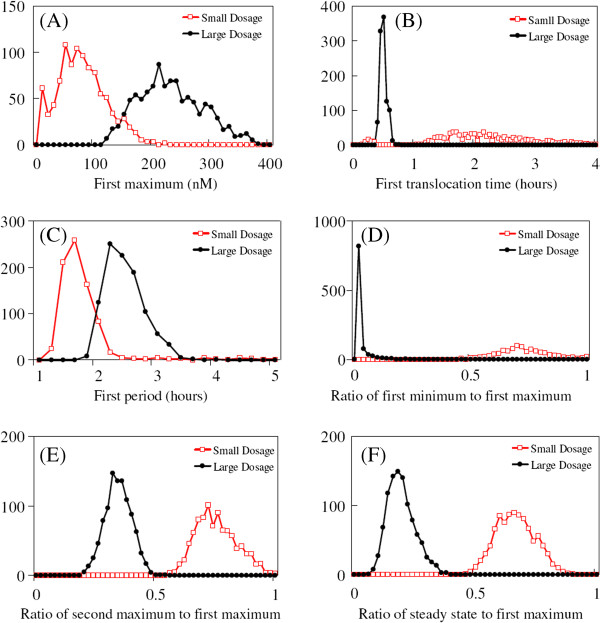
**Distributions of six dynamic features of nuclear NF-κB profiles for an ensemble of 1,000 replicates of the wild type NF-κB signaling system undergoing small (TR = 0.01; red line) or large (TR = 1; black line) dosage stimulations.** The six dynamic features are First Maximum in panel (**A**), First Translocation Time in panel (**B**), First Period in panel (**C**), Ratio of First Minimum to First Maximum in panel (**D**), Ratio of Second Maximum to First Maximum in panel (**E**), and Ratio of Steady State to First Maximum in panel (**F**).

The distributions of responses for a thousand-replicate ensemble to large (TR = 1) and small (TR = 0.01) dosage are shown in Figure 10. In Figure 10(A) and (C), both the First Maximum and the First Period share similar dosage-dependent behavior: the strength and duration of the response increase with dosage. But, for the First Translocation Time and the Ratio of the First Minimum to the First Maximum metrics in Figure 10(B) and (D), the dosage-dependent behavior is inverted: the larger dosage induces a peak at an earlier time with a smaller First Minimum level. Moreover, the larger dosage makes the distribution more narrowly-distributed. This indicates the larger dosage induces an earlier and spikier response, while the smaller dosage induces more heterogeneous First Maximum and First Minimum levels of nuclear NF-кB concentration. Lastly, both the ratios of the Second Maximum to the First Maximum and of the Steady State to the First Maximum share similar dosage-dependent behavior in Figures 10(E) and 10(F): the smaller dosage induces a distribution at larger values, i.e., closer to one. In other words, when stimulated by the smaller dosage, the levels of the First Maximum, of the subsequent maxima, and of the Steady State are the same, i.e., NF-кB profiles exhibit either a monotonically-increasing pattern or single-peaked pattern with low peak amplitude. In addition, the full half-maximum width of the distribution is unaffected by the dosage amount.

#### Dosage-dependent distribution of the dynamic patterns

**Figure 11:**
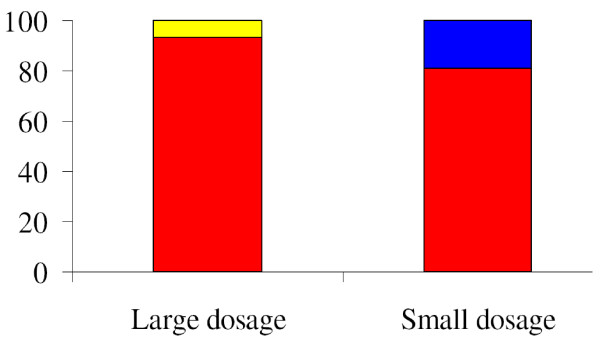
**Distribution of the dynamic patterns of nuclear NF-кB concentration profiles for an ensemble of 1,000 replicates of the wild type NF-кB signaling system undergoing small (TR = 0.01) or large (TR = 1) dosage stimulations.** The same four dynamic patterns and coloring scheme are used as in Figure 6.

As shown in Figure 11, when stimulated by a small (TR = 0.01) dosage, 80% of the nuclear NF-кB profiles are damped-oscillatory whereas only 20% of are single-peaked. But, those damped oscillatory responses are similar to a single-peaked response. The distribution induced by the large dosage (TR = 1) corresponds to that of the wild type case in Figure 6. We note that for small dosage stimulation the distribution of the dynamic patterns, the SE average, and the individual profiles of nuclear NF-кB concentration, as shown in Figures 9 and 11, are very similar to those for the mutants responding to large dosage stimulation with IкBi and IкBϵ genes double knocked-out, as shown in Figures 5 and 6. We also observed that when the heterogeneity factor χ is increased from χ = 30% to χ = 70%, small dosage stimulation generates more heterogeneous dynamic patterns, i.e. nuclear NF-кB profiles in all the pattern categories.

In this subsection, we analyzed the dynamical response of the cellular replicates under two different stimulant dosage conditions. This yielded distributions of six dynamic features and associated dynamic patterns that are descriptive characterizations of the NF-кB signaling system. Unlike the earlier analysis of differential genetic conditions, the differing stimulus dosages generate non-overlapping distributions and clearly distinctive dynamical behaviors. Some of our predictions, e.g., the distribution of first translocation time in Figure 10(B) and dynamic patterns in Figure 11, are experimentally validated [36].

### Sigmoidally shaped SE average of the dose-response curves

**Figure 12:**
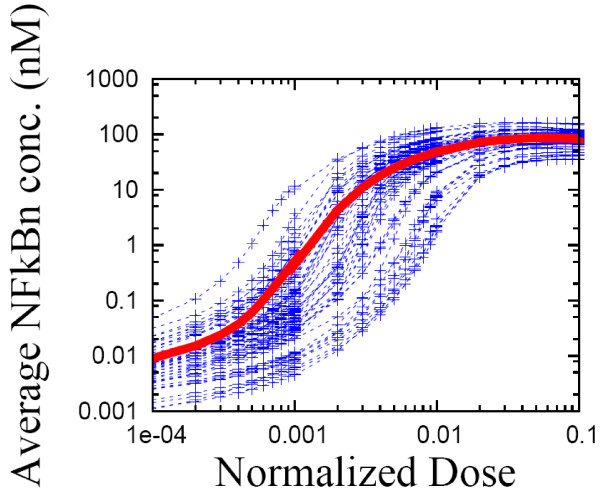
The individual dose-response curves (blue lines) and the statistical ensemble average (red line) for an ensemble of 50 replicates of the wild type NF-кB signaling system.

We numerically investigated the distribution of dose-response curves from the SE of the NF-кB system. In this analysis we used only 50 replicates of the NF-кB system because of the high computational cost of calculating a single dose-response curve. Each replicate of the NF-кB signaling system is stimulated with a persistent signal for 30 hours, and the average (quasi-steady-state) level of nuclear NF-кB concentration is measured between 20 and 30 hours after stimulation. To check for hysteresis effects, we computed the dose response curve twice, first by increasing the signal strength from *TR = 0* to *TR = 0.1* in a step-like manner and then by decreasing it from *TR = 0.1* to *TR = 0.* If the forward and backward dose-response curves were significantly different, it could be regarded as a sign of hysteresis. In Figure 12, both forward and backward dose-response curves for each replicate look the same, i.e. no hysteresis effects were seen. Figure 12 also shows both the individual dose-response curves and the SE average have a sigmoidal shape, which indicates a switching behavior between on and off states. For a signal strength greater than the inflection point of a dose-response curve, the stationary level quickly reaches a plateau whose value is three orders of magnitude higher than its low stationary level at a signal smaller than the inflection point. Lastly, the cell-to-cell variability in the stationary nuclear NF-кB level is dramatically larger with a weak signal than with a strong signal, i.e., the variation is two orders of magnitude at *TR = 10*^*-4*^ and one order of magnitude at *TR = 0.1*. The variability is a maximum at the inflection point of the SE dose-response curve, where it is roughly four orders of magnitude. The cell-to-cell variability in the location of the inflection point (along the x-axis) is about one order of magnitude.

## Discussion and conclusion

In this paper, we have used a novel statistical ensemble (SE) method to mimic protein dynamics in a population of cells influenced by extrinsic noise. For our model of the NF-кB signaling system, we showed that the SE averages match population-averaged experimental data. The added value of the SE method is that it can also produce entire distributions of response, which can potentially be compared to experimental observations at the single-cell level. The main predictions enabled by the SE method were as follows: (a) nuclear NF-кB concentration profiles for single cells are expected to fall into one of several distinct heterogeneous dynamic patterns, (b) larger dosages should induce more oscillatory dynamic patterns of nuclear NF-кB response, while smaller dosages should primarily induce single-peaked patterns, (c) larger (smaller) dosages should make First translocation times more narrowly-distributed (broadly-distributed) and shift the peak of its distribution to earlier (later) times, and (d) the shape of dose-response curves, both at the single-cell and population level, should be sigmoidal. After making these predictions computationally, our experimental colleagues used single-cell fluorescence imaging to monitor NF-кB nucleo-cytoplasmic translocation dynamics in lipopolysaccharide-insulted murine macrophage cells, and found that nuclear GFP-RelA (a subunit of NF-кB dimers) profiles are very heterogeneous. They also found NF-кB dynamic responses to be much more heterogeneous and less oscillatory when the stimulant dosage was smaller. They also stimulated the murine macrophages with two different dosages (1 nM and 1 μM) of E. Coli lipopolysaccharide and found two distinctly different distributions of NF-кB translocation time [36]. Thus two of our predictions have been verified by our collaborators who are planning to publish the results elsewhere. We hope our computational analyses will elicit more single-cell experimental measurement to verify the predicted dynamic behaviors.

We wish to emphasize that the novelty of our analysis is not due to its methodology, but rather the viewpoint we adopt with regard to computational modeling of cellular response. Most previous modeling efforts have focused on bifurcation analysis of the response of a dynamical system as its input kinetic rate constants are varied. This approach makes a one-to-one correspondence between a form of dynamic response and a single set of parameter values. By contrast, the assumption in the SE approach is that the dynamics of protein response in individual cells is intrinsically heterogeneous. We assume a population of cells (the replicates of the SE) does not occupy a single point, but rather a volume of points in high-dimensional parameter space. We choose a hybercube sub-volume of this space (a midpoint and interval size for each dimension) and sample from it efficiently to assign kinetic parameters to each replicate in the SE. In this regard, our SE approach looks similar to sensitivity analysis by using Latin Hypercube Sampling method. But, our analysis is not for sensitivity analysis of cell signaling systems to perturbation of model parameter values, but for generation of the heterogeneous responses in single cells. As explained below, we choose the bounds of this sub-volume by fitting the resulting SE averages to experimentally observed population-level averages. Once this is done and the averages match, our assumption is that we can understand the heterogeneous behavior of the biochemical network at the single-cell level by analyzing the wealth of distribution data provided by the SE computations across its set of replicates.

The sigmoidal shape of the dose-response curve reveals two important properties of NF-кB signaling: its switching behavior and its monostability (i.e., no hysteresis). The inflection points of individual sigmoidal curves can be viewed as activation thresholds for the NF-кB signaling pathway. As shown in Figure 12, the NF-кB response is quite small for signal strength below the threshold, while the response increases dramatically (log scale on y-axis) for signal strengths just above the threshold. Knowing that some NF-кB target genes are inflammatory cytokines and that over-expressed inflammatory response is harmful to the host, we can speculate that the NF-кB signaling network employs this sigmoidal dose-response curve to down-regulate excessive inflammatory responses, i.e., to only turn on if the danger level is significantly high, otherwise to shut down. We also note that the amplitude and timing of the first response peak for inflammatory cytokines (such as TNFα) are known to be critical in mediating timely and effective immune response. This is motivation for measuring the dosage-dependent transient dynamic response of NF-κB target genes to investigate the shape of the dose-response. Lastly, TNFα autocrine signaling forms a positive feedback loop in the NF-кB signaling network and can induce bistability, which could modify our results indicating monostability.

Our statistical analysis of protein dynamics depends on how accurately the computationally generated ensemble of the NF-кB signaling system represents a true biological population of individual cells. This question is equivalent to what is the true distribution of extrinsic noise? I.e., what is the distribution from which the kinetic input parameters should be sampled? In this paper, we have chosen a simple answer to this question by assuming the distribution is uniform and bounded. We devised a heuristic fitting algorithm to find the bounding limits of the uniform distribution for each kinetic parameter by minimizing the discrepancy between the SE averages and the population-level experimental data. This heuristic scheme could be converted to a more rigorous optimization problem: to find the distribution of kinetic parameters for a network model which minimizes the difference between the SE average and the population-level experimental measurements, while simultaneously reproducing the range of experimentally-observed heterogeneous protein dynamics in single cells.

Additional improvements could also be made to the procedure for sampling from the parameter space. For example, the sampling could become more biologically relevant, by accounting for changes in the distribution of extrinsic noise over time as cells traverse their cell cycle. We have also assumed no correlation between pairs of kinetic parameters. In fact some parameters may be co-dependent because cellular energy resources are limited: e.g., as one kinetic process is accelerated, others may be inhibited to balance cellular energy consumption. All of these computational tasks would be made easier with additional single-cell experimental data from which the true distribution of extrinsic noise could be inferred.

Finally, we note that our analysis in this paper was simplified by categorizing the nuclear NF-кB response profiles into four dynamic patterns. This simplified various statistical analyses and made it easier to characterize changes in the distribution when genes were knocked-out. Our choice was based on mathematical characterization of the dynamic protein profiles. However, it is possible this neglects other biologically important details of the nuclear NF-кB response, e.g. classification by time periodicity or by steady-state level. Since the choice of categories can affect subsequent analysis, this is an important factor to consider when using the SE methodology.

## Methods

### Six dynamic features of nuclear NF-кB profiles

We define six dynamic features to represent the distinguishing characteristics of temporal profiles of nuclear NF-кB concentration. The first translocation time is the time when the first peak occurs; the first period measures the time between the first two peaks; the first and second maxima are the amplitudes of the first and second peaks; the first minimum is the amplitude of the “valley” between the first two peaks; steady state refers to the asymptotic amplitude at sufficiently long time. Using the first maximum as a reference level, we use scaled ratios, i.e., the first minimum, the second maximum, and the steady state are normalized by the first maximum. The distributions of these dynamical features are presented in Figures 8 and 10.

### Generation of the SE of NF-кB signaling network

Each kinetic rate constant listed in Table 1 is randomly sampled from an interval (*x*_*0*_*(1-χ), x*_*0*_*(1 + χ)*) where *χ*_0_ is the reference value and *χ* is a heterogeneity factor. To sample efficiently in the high dimensional space of dozens of parameters, we use the Latin Hypercube Sampling methodology discussed below. For this paper, we used *χ* = 0.3. To generate a statistical ensemble (SE) of *N* replicates, we simply generate *N* sets of randomly sampled kinetic parameters.

### Algorithm to fit the SE average to population-level experimental data

The goal of our fitting algorithm is to determine kinetic parameters that provide the best match for features of the SE average to the experimental time-series data. We do not attempt to fit all of the eighty kinetic parameters. This can result in “over-fitting”, with too many parameters fit to too little data. Also, by sensitivity analysis, others and we have found there are only a handful of kinetic parameters in the NF-кB signaling network whose variation significantly affects the temporal profile of the nuclear NF-кB concentration [23,35,40]. Based on our previous studies [35,40], we choose the two kinetic parameters most highly correlated with each dynamic feature and varied that set of parameters in the fitting procedure. The heuristic steps are as follows. (1) Use an educated guess for initial kinetic parameters and set the heterogeneity factor to *χ* = 039. (2) Generate the SE and resulting protein profiles and calculate the deviation of the six dynamic features of the SE average from the target (experimental) dynamic features. (3) Identify the dynamic feature with largest deviation and modify the two kinetic parameters associated with it. (4) Repeat steps 1-3 until the dynamic features are close to the target values. (5) When a good fit is not achievable decrease *χ* in a step-like manner. All of the data in Figures 3, 5, 4, 7 were obtained through this process.

### Numerical simulation of the NF-кB signaling network

A coupled system of ordinary differential equations (ODEs) is derived from the NF-кB signaling network in Figure 2. Using a 4^th^ order Runge-Kutta scheme, we numerically integrate the ODEs, using initial conditions (i.e., the cytoplasmic NF-кB concentration being equal to the total NF-кB concentration and zero concentrations of all other biochemical species) and kinetic parameters shown in Table 1 and sampled as described below. Before stimulation, the system runs for 33 hours until its constituents reach equilibrium values. Then, we simulate persistent stimulation by turning on the reaction, IKKn → IKKa with a rate TR*K1 and a non-zero constant value of TR. The ChemCell software package is used to carry out part of numerical simulation [41].

### Latin hypercube sampling (LHS)

LHS is a constrained Monte Carlo sampling scheme. Monte Carlo sampling is a commonly-used approach for assessing the uncertainty of a computational model. By sampling repeatedly from the assumed joint probability function of the input variables, and evaluating the response for each sample, the distribution of responses of the model can be estimated. This approach yields reasonable estimates for the distribution of responses, but a large number of samples is needed if there are many input variables, i.e. a high-dimensional input space. A large sample size can be computationally expensive, which motivates an alternative approach, namely LHS. LHS yields more precise estimates of the response distribution with a smaller number of samples from high-dimensional input spaces [42]. Suppose that the model has K kinetic rate variables and we want N samples, where a sample is a set of K values, one per variable. LHS first selects N different values for each of the K variables, by dividing the range of each variable into N non-overlapping intervals on the basis of equal probability. One value from each interval is selected randomly, in accord with the assumed probability density within the interval. The N values for the first kinetic rate variable are then paired in a random manner (equally likely combinations) with the N values of the second variable. These N pairs are combined in a random manner with the N values of the third variable to form N triplets, and so on, until N K-tuples are formed. Each K-tuple becomes a set of kinetic rate parameters for one replicate within the statistical ensemble of N replicates.

## Competing interests

The authors declare that they have no competing interests.

## Authors’ contributions

JJ conceived and designed the numerical experiments and analyzed the simulation results. JJ and SJP developed code specific for these models and performed the numerical experiments. JJ wrote the paper and SJP and JLF edited the paper. All authors read and approved the final manuscript.

## References

[B1] Thattai M van Oudenaarden A Intrinsic noise in gene regulatory networks Proc Natl Acad Sci 2001 98 8614 8619 10.1073/pnas.151588598 11438714 PMC37484

[B2] Elowitz MB Levine AJ Siggia ED Swain PS Stochastic gene expression in a single cell Science 2002 297 1183 1186 10.1126/science.1070919 12183631

[B3] Swain PS Elowitz MB Siggia ED Intrinsic and extrinsic contributions to stochasticity in gene expression Proc Natl Acad Sci 2002 99 12795 12800 10.1073/pnas.162041399 12237400 PMC130539

[B4] Blake WJ Karn M Cantor CR Collins JJ Noise in eukaryotic gene expression Science 2003 422 633 637 10.1038/nature01546 12687005

[B5] Raser JM O’Shea EK Control of stochasticity in eukaryotic gene expression Science 2004 304 1811 1814 10.1126/science.1098641 15166317 PMC1410811

[B6] Rosenfeld N Young JW Alon U Swain SS Elowitz MB Gene regulation at the single-cell level Science 2005 307 1962 1965 10.1126/science.1106914 15790856

[B7] Pedraza JM van Oudenaarden A Noise propagation in gene networks Science 2005 307 1965 1969 10.1126/science.1109090 15790857

[B8] Raj A Perskin CS Tranchina D Vargas DY Tyagi S Stochastic mRNA synthesis in mammalian cells PLoS Biol 2006 4 e309 10.1371/journal.pbio.0040309 17048983 PMC1563489

[B9] Cohen AA Geva-Zatorsky N Eden E Frenkel-Morgenstern M Issaeva I Sigal A Milo R Cohen-Saidon C Liron Y Kam Z Cohen L Danon T Perzov N Alon U Dynamic proteomics of individual cancer cells in response to a drug Science 2008 322 1511 1516 10.1126/science.1160165 19023046

[B10] Spencer SL Gaudet S Albeck JG Burke JM Sorger PK Non-genetic origins of cell-to-cell variability in TRAIL-induced apoptosis Nature 2009 459 428 432 10.1038/nature08012 19363473 PMC2858974

[B11] McAdams HH Shapiro L Circuit simulation of genetic networks Science 1995 269 650 656 10.1126/science.7624793 7624793

[B12] Arkin A Ross J McAdams HH Stochastic kinetic analysis of developmental pathways bifurcation in phage λ-infected E. Coli cells Genetics 1998 149 1633 1648 9691025 10.1093/genetics/149.4.1633 PMC1460268

[B13] Blake WJ Balazsi G Kohanski MA Isaacs FJ Murphy KF Kuang Y Cantor CR Walt DR Collins JJ Phenotypic consequences of promoter-mediated transcriptional noise Mol Cell 2006 24 853 865 10.1016/j.molcel.2006.11.003 17189188

[B14] Paulsson J Summing up the noise in gene networks Nature 2004 427 415 418 10.1038/nature02257 14749823

[B15] Shahrezaei V Ollivier JF Swain PS Colored extrinsic fluctuations and stochastic gene expression Mol Syst Biol 2008 4 196 18463620 10.1038/msb.2008.31 PMC2424296

[B16] Brown KS Hill CC Calero GA Myers CR Lee KH Sethna JP Cerione RA The statistical mechanics of complex signaling networks: nerve growth factor signaling Phys Biol 2004 1 184 195 10.1088/1478-3967/1/3/006 16204838

[B17] Huang K Statistical Mechanics 1987 second New York: Wiley

[B18] Verma IM Stevenson J IκB kinase: beginning, not the end Proc Natl Acad Sci U S A 1997 94 11758 11760 10.1073/pnas.94.22.11758 9342307 PMC33775

[B19] Li Q Verma IM NF-κB regulation in the immune system Nat Rev Immunol 2002 2 725 10.1038/nri910 12360211

[B20] Hoffmann A Baltimore D Circuitry of nuclear factor κB signaling Immunol Rev 2006 210 171 186 10.1111/j.0105-2896.2006.00375.x 16623771

[B21] Hoffmann A Levchenko A Scott ML Baltimore D The IκB-NF-κB signaling module: temporal control and selective gene activation Science 2002 298 1241 1245 10.1126/science.1071914 12424381

[B22] Lee EG Boone DL Chai S Libby SL Chien M Lodolce JP Ma A Failure to regulate TNF-induced NF-κB and cell death responses in A20-deficient mice Science 2000 289 2350 2354 10.1126/science.289.5488.2350 11009421 PMC3582399

[B23] Lipniacki T Paszek P Brasier AR Luxon BA Kimmel M Mathematical model of NF-κB regulatory module J Theor Biol 2004 228 195 215 10.1016/j.jtbi.2004.01.001 15094015

[B24] Lipniacki T Paszek P Brasier AR Luxon BA Kimmel M Stochastic regulation in early immune response Biophys J 2006 90 725 742 10.1529/biophysj.104.056754 16284261 PMC1367099

[B25] Nelson DE Ihekwaba AEC Elliott M Johnson JR Gibney CA Foreman BE Nelson G See V Horton CA Spiller DG Edwards SW McDowell HP Unitt JF Sullivan E Grimley R Benson N Broomhead D Kell DB White MRH Oscillations in NF-κB signaling control the dynamics of gene expression Science 2004 306 704 708 10.1126/science.1099962 15499023

[B26] Nelson DE Horton CA See V Johnson JR Nelson G Spiller DG Response to comments on “oscillations in NF-κB signaling control the dynamics of gene expression” Science 2005 308 52b 10.1126/science.110790415802586

[B27] Barken D Wang CJ Kearns J Cheong R Hoffmann A Levchenko A Comment on “oscillations in NF-κB signaling control the dynamics of gene expression” Science 2005 308 52a 10.1126/science.1107904 15802586 PMC2821939

[B28] Lee TK Denny EM Sanghvi JC Gaston JE Maynard ND Hughey JJ Covert MW A noisy paracrine signal determinines the cellular NF-κB response to lipopolysaccharide Sci Signal 2009 2 93 ra 65 10.1126/scisignal.2000599 PMC2778577 19843957

[B29] Ashall L Horton CA Nelson DE Paszek P Harper CV Sillitoe K Ryan S Spiller DG Unitt JF Broomhead DS Kell DB Rand DA Sée V White MR Pulsatile stimulation determines timing and specificity of NF-κB -dependent transcription Science 2009 324 242 246 10.1126/science.1164860 19359585 PMC2785900

[B30] Bartfeld S Hess S Bauer B Machuy N Ogilvie LA Schuchhardt J Meyer TF High-throughput and single-cell imaging of NF-κB oscillations using monoclonal cell lines BMC Cell Biol 2010 11 21 10.1186/1471-2121-11-21 20233427 PMC2848210

[B31] Turner DA Paszek P Woodcock DJ Nelson DE Horton CA Wang Y Spiller DG Rand DA White MRH Harper CV Physiological levels of TNF stimulation induce stochastic dynamics of NF-κB responses in single living cells J Cell Sci 2010 123 2834 2843 10.1242/jcs.069641 20663918 PMC2915884

[B32] Tay S Hughey JJ Lee TK Lipniacki T Quake SR Covert MW Single-cell NF-κB dynamics reveal digital activation and analogue information processing Nature 2010 466 267 271 10.1038/nature09145 20581820 PMC3105528

[B33] Paszek P Ryan S Ashall L Sillitoe K Harper CV Spiller DG Rand DA White MR Population robustness arising from cellular heterogeneity Proc Natl Acad Sci 2010 107 11644 11649 10.1073/pnas.0913798107 20534546 PMC2895068

[B34] Hayot F Jayaprakash C NF-κB oscillations and cell-to-cell variability J Theor Biol 2006 240 583 591 10.1016/j.jtbi.2005.10.018 16337239

[B35] Ihekwaba AEC Broomhead DS Grimley RL Benson N Kell DB Sensitivity analysis of parameters controlling oscillatory signaling in the NF-κB pathway: the roles of IKK and IκBα Syst Biol 2004 1 93 103 10.1049/sb:20045009 17052119

[B36] James CD Moorman MW Carson BD Branda CS Lantz JW Manginell RP Martino A Singh AK Nuclear translocation kinetics of NF-κB in macrophages challenged with pathogens in a microfluidic platform Biomed Microdevices 2009 11 693 700 10.1007/s10544-008-9281-5 19169824

[B37] Tian B Nowak DE Brasier AR A TNF-induced gene expression program under oscillatory NF-κB control BMC Genomics 2005 6 137 155 10.1186/1471-2164-6-137 16191192 PMC1262712

[B38] Werner SL Barken D Hoffmann A Stimulus specificity of gene expression programs determined by temporal control of IKK activity Science 2005 309 1857 1861 10.1126/science.1113319 16166517

[B39] Covert MW Leung TH Gaston JE Baltimore D Achieving stability of lipopolysaccharide-induced NF-κB activation Science 2005 309 1854 1857 10.1126/science.1112304 16166516

[B40] Joo J Plimpton S Martin S Swiler L Faulon JL Sensitivity analysis of a computational model of the IKK-NF-kappaB-IkappaBalpha-A20 signal transduction network Ann NY Acd Sci 2007 1115 221 239 10.1196/annals.1407.014 17934057

[B41] Plimpton SJ Slepoy A Microbial cell modeling via reacting diffusing particles J Phys: Conference Series 2005 16 305 309

[B42] Swiler LP Wyss GD A user’s guide to Sandia’s Latin Hypercube sampling software: LHS UNIX library/standalone version SAND Report 2004 2004 243923802142

